# Novel Nonnucleoside Inhibitors of Zika Virus Polymerase Identified through the Screening of an Open Library of Antikinetoplastid Compounds

**DOI:** 10.1128/AAC.00894-21

**Published:** 2021-08-17

**Authors:** Yanira Sáez-Álvarez, Nereida Jiménez de Oya, Carmen del Águila, Juan-Carlos Saiz, Armando Arias, Rubén Agudo, Miguel A. Martín-Acebes

**Affiliations:** a Facultad de Farmacia, Universidad CEU San Pablo, Madrid, Spain; b Department of Biotechnology, Instituto Nacional de Investigación y Tecnología Agraria y Alimentaria, Consejo Superior de Investigaciones Científicas (INIA, CSIC), Madrid, Spain; c Unidad de Medicina Molecular, Centro Regional de Investigaciones Biomédicas, Universidad de Castilla-La Mancha (UCLM), Albacete, Spain

**Keywords:** Zika virus, West Nile virus, dengue virus, polymerase, nonnucleoside inhibitor, allosteric, antiviral, RNA polymerases, antiviral agents

## Abstract

Zika virus (ZIKV) is a mosquito-borne pathogen responsible for neurological disorders (Guillain-Barré syndrome) and congenital malformations (microcephaly). Its ability to cause explosive epidemics, such as that of 2015 to 2016, urges the identification of effective antiviral drugs. Viral polymerase inhibitors constitute one of the most successful fields in antiviral research. Accordingly, the RNA-dependent RNA polymerase activity of flavivirus nonstructural protein 5 (NS5) provides a unique target for the development of direct antivirals with high specificity and low toxicity. Here, we describe the discovery and characterization of two novel nonnucleoside inhibitors of ZIKV polymerase. These inhibitors, TCMDC-143406 (compound 6) and TCMDC-143215 (compound 15) were identified through the screening of an open-resource library of antikinetoplastid compounds using a fluorescence-based polymerization assay based on ZIKV NS5. The two compounds inhibited ZIKV NS5 polymerase activity *in vitro* and ZIKV multiplication in cell culture (half-maximal effective concentrations [EC_50_] values of 0.5 and 2.6 μM for compounds 6 and 15, respectively). Both compounds also inhibited the replication of other pathogenic flaviviruses, namely, West Nile virus (WNV; EC_50_ values of 4.3 and 4.6 μM for compounds 6 and 15, respectively) and dengue virus 2 (DENV-2; EC_50_ values of 3.4 and 9.6 μM for compounds 6 and 15, respectively). Enzymatic assays confirmed that the polymerase inhibition was produced by a noncompetitive mechanism. Combinatorial assays revealed an antagonistic effect between both compounds, suggesting that they would bind to the same region of ZIKV polymerase. The nonnucleoside inhibitors of ZIKV polymerase here described could constitute promising lead compounds for the development of anti-ZIKV therapies and, eventually, broad-spectrum antiflavivirus drugs.

## INTRODUCTION

Zika virus (ZIKV) is a mosquito-borne pathogen from the genus *Flavivirus* (family *Flaviviridae*). This virus is transmitted by the bites of *Aedes* mosquitoes, but it can also be sexually or vertically acquired. Most infections are asymptomatic or induce a mild illness characterized by rash, low-grade fever, arthralgia and myalgia, or conjunctivitis. However, in some cases, the infection courses with neurological manifestations (Guillain-Barré syndrome) (1). Mother-to-child transmission can result in congenital Zika syndrome characterized by a broad spectrum of fetal and birth defects, including microcephaly (2). This pathogen was historically associated with sporadic outbreaks or regional epidemics of mild disease in tropical Africa, South Asia, and the Pacific. However, in 2016, a public health emergency of international concern was declared by the World Health Organization due to the rapid expansion of ZIKV throughout Latin America and the Caribbean (3). The number of cases declined during the subsequent years, but due to the ability of the virus to cause explosive epidemics, there is still a real threat of (re)emergence of ZIKV in the world (4, 5).

The ZIKV genome is a single RNA molecule of positive polarity, about 11 kb in length, that encodes a single open reading frame. The genome is translated into a polyprotein further processed by cellular and viral proteases to produce three mature structural proteins (C, M, and E) and seven nonstructural proteins (NS1, NS2A, NS2B, NS3, NS4A, NS4B, and NS5). Only NS3 and NS5 have characterized enzymatic activities. NS3 functions as the viral protease and also harbors an RNA helicase domain, necessary for unwinding genomic RNA during replication (6). NS5 is a highly conserved protein among all flaviviruses and comprises two domains, an N-terminal methyltransferase domain and a C-terminal RNA-dependent RNA polymerase (RdRp) domain (7, 8). The methyltransferase activity of NS5 is related to the formation of the 5′ cap structure of the viral RNA, involved in both the translation and the evasion of the host's innate immune response (9, 10), whereas the RdRp activity is responsible for viral genome replication. The three-dimensional structure of the ZIKV NS5 RdRp domain exhibits high similarities with the rest of the flavivirus RdRps, showing the characteristic encircled right-hand configuration with “fingers,” “palm,” and “thumb” subdomains. The fingers and the thumb share extensive interactions that surround the palm, where the conserved motifs (A to E), which are common to other RdRps, are located. These motifs are crucial for the polymerase activity of RdRps since they are involved in RNA and nucleotide binding and nucleotidyl transfer reaction (11). In addition, the structure shows a conserved extension of the thumb subdomain called the “priming loop” that is the hallmark of the RdRps from *Flavivirus* and enables these polymerases to perform *de novo* initiation of RNA synthesis (12). Thus, the unique nature of ZIKV NS5 RdRps makes this enzyme one of the most attractive targets for antiviral discovery (13–16).

The current lack of vaccines or specific therapies against ZIKV urges identification of new tools that can help to fight future epidemics, such as the use of nucleoside or nonnucleoside polymerase inhibitors (NIs or NNIs, respectively). NIs mimic natural polymerase substrates, leading to termination of elongation upon incorporation into the nascent nucleic acid chain. NNIs bind allosteric sites of the polymerase away from its active center, inhibiting its activity through the induction of conformational changes. As viral polymerase inhibitors constitute one of the most promising antiviral therapeutic approaches (17), here, we leverage an established fluorescence-based method to measure ZIKV NS5 RdRp activity (18) as a high-throughput screening assay for the identification and characterization of novel ZIKV RdRp inhibitors with antiviral activity.

## RESULTS

### Library screening.

**FIG 1:**
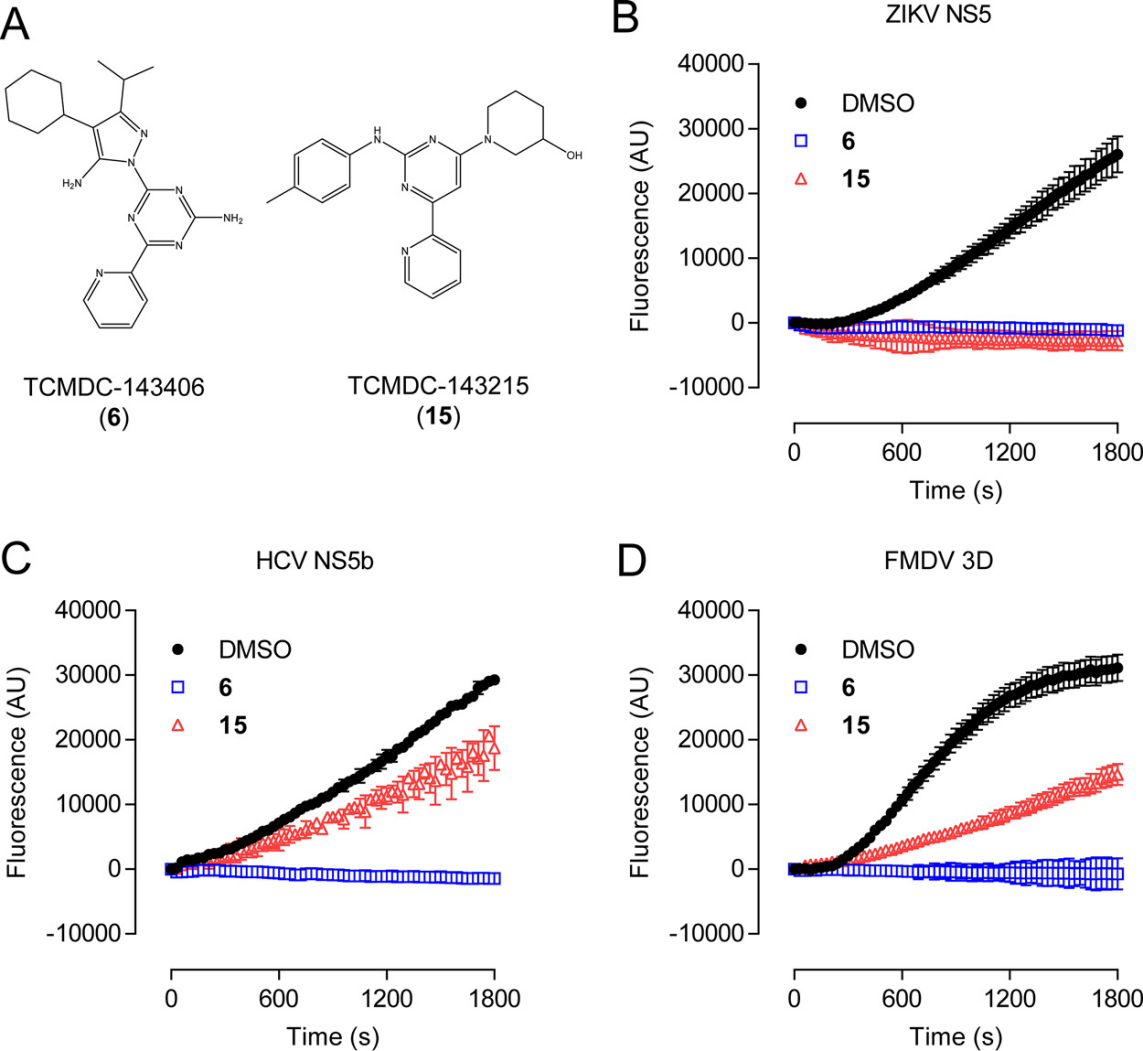
Novel ZIKV polymerase inhibitors. (A) Structure of compounds 6 and 15. (B) Real-time fluorescence-based enzymatic activity of ZIKV NS5 RdRp in the absence (black circles) or in the presence of 100 μM compound 6 (blue squares) or 100 μM compound 15 (red triangles). Fluorometric measurements were performed as described in Materials and Methods (*n *= 4). (C and D) Same as in panel A, but using HCV NS5b and FMDV 3D RdRps, respectively (*n *= 4). Data are expressed as mean ± SD.

In the search for novel inhibitors of ZIKV NS5 RdRp, the high-throughput fluorescence-based method previously designed by us was used as a screening platform (18). To this aim, the open-access chemical library against kinetoplastids from GlaxoSmithKline (GSK) (19) was subjected to analysis. This library was selected for several factors as a source of chemicals. First, its open-source format makes it easy to access for screening; second, it comprises a collection of compounds filtered for nonspecific cytotoxicity; and finally, the potential to identify compounds with both antiparasitic and antiviral activities constitutes an attractive goal to push the development of therapies against neglected infectious diseases. All compounds were tested at a final assay concentration of 100 μM by real-time fluorescence-based polymerization assays using a 96-well format according to the protocol previously developed (18). Only compounds that exhibited total inhibition of the fluorescence-associated activity compared to a dimethyl sulfoxide (DMSO) control during the whole course of the reaction were considered hits. From the initial screening of 592 compounds, two potential polymerase inhibitors were identified, TCMDC-143406 (compound 6) and TCMDC-143215 (compound 15) (Fig. 1A and B). Levering the versatility of this method to be exploited with other systems (18), the effect of compounds 6 and 15 was also assessed against other RdRps. We selected the NS5b RdRp from hepatitis C virus (HCV) (Fig. 1C), which belongs to the genus *Hepacivirus* (family *Flaviviridae*) (Fig. 1C), and the 3D RdRp from the more genetically distant *Aphthovirus* (family *Picornaviridae*) foot-and-mouth disease virus (FMDV) (Fig. 1D). Compound 6 totally inhibited the polymerization reaction of both HCV and FMDV RdRps. In contrast, compound 15 showed only partial inhibition of HCV NS5b and FMDV 3D (Fig. 1C and D). Overall, these results indicate that compounds 6 and 15 could represent interesting lead compounds for the development of ZIKV RdRp inhibitors.

### *In vitro* potency and mechanism of inhibition.

**FIG 2:**
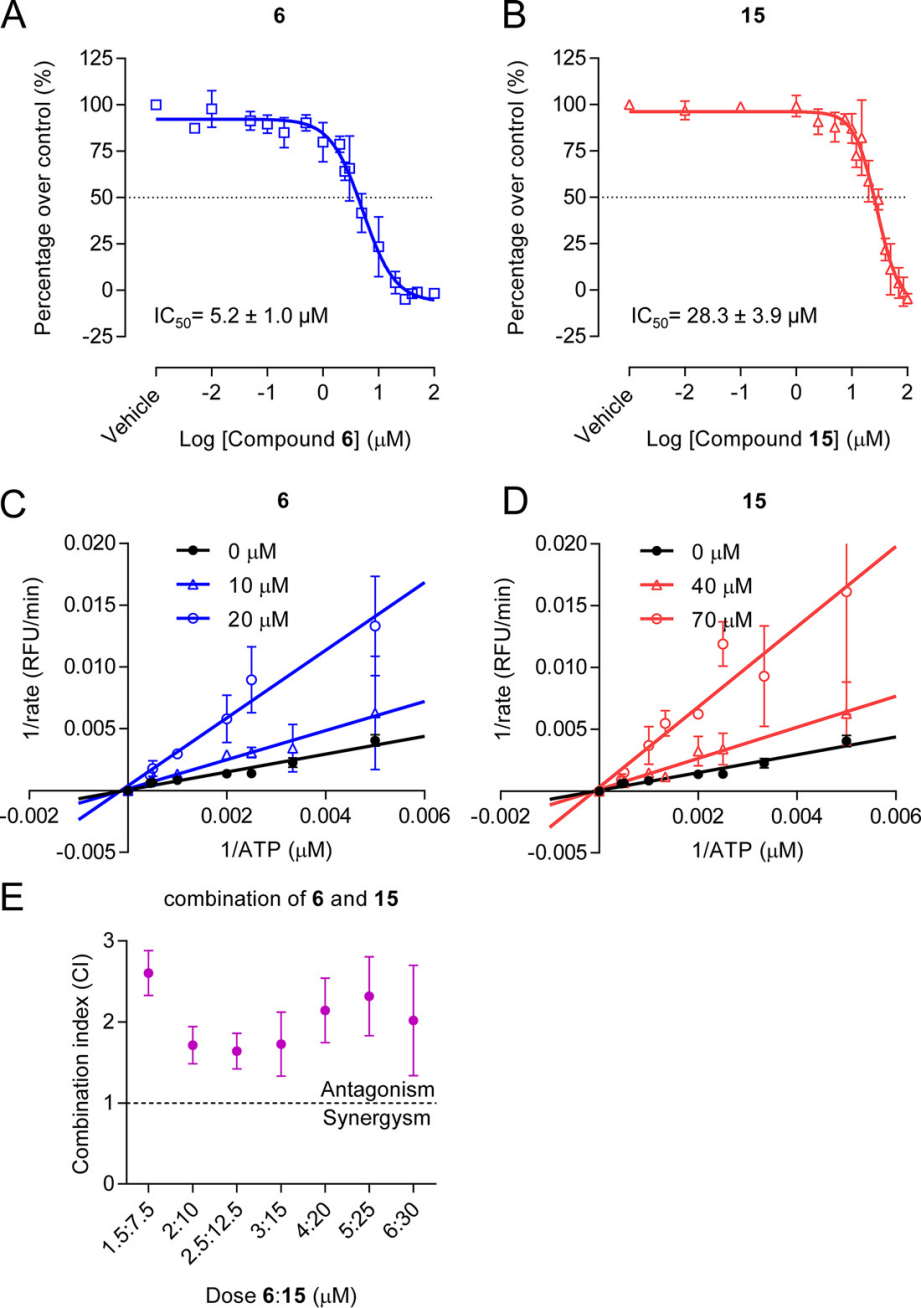
Biochemical characterization of polymerase inhibitors. (A and B) Dose-response curves of ZIKV RdRp against compound 6 (A) and compound 15 (B) (*n *= 4). (C and D) Enzyme inhibition kinetics of compound 6 (C) and compound 15 (D) against ZIKV RdRp. Fluorescence-based polymerization assays were performed using increasing concentrations of either compound 6 or 15 (*n *= 3). Experimental conditions, compounds concentrations, and data processing are explained in the corresponding section of Materials and Methods. (E) Combination study between compounds 6 and 15. The combination index (CI) plot of different combination doses of compounds 6 and 15 (*n *= 4). Data above or below the dotted line (CI = 1) represent antagonism and synergism, respectively. Data are expressed as mean ± SD.

To evaluate the potency of compounds 6 and 15, the half-maximal inhibitory concentrations (IC_50_s) of the compounds were calculated (Fig. 2A and B). Both compounds were active in the micromolar range, with compound 6 about 5-fold more potent than compound 15 (IC_50_s of 5.21 μM and 28.32 μM for compounds 6 and 15, respectively). In an effort to gain insight into the mechanism of inhibition, the velocity of reaction carried out in the presence of increasing concentrations of substrate ATP and either compounds 6 or 15 was analyzed (Fig. 2C and D). Results from Lineweaver-Burk plots of compounds 6 and 15 were indicative of noncompetitive inhibition. Next, the potential interactions between the compounds were addressed by means of combinatorial assays, and the combinatory indexes (CIs) were calculated (Fig. 2E). In these experiments, CI values of <1, 1, or >1 would mean synergistic, additive, or antagonistic activity, respectively (20). The CI of >1 observed in all cases suggested that the compounds exerted antagonistic activity between them, which could reflect similarities in their mechanism of inhibition.

### Antiviral activity against pathogenic flaviviruses.

**FIG 3:**
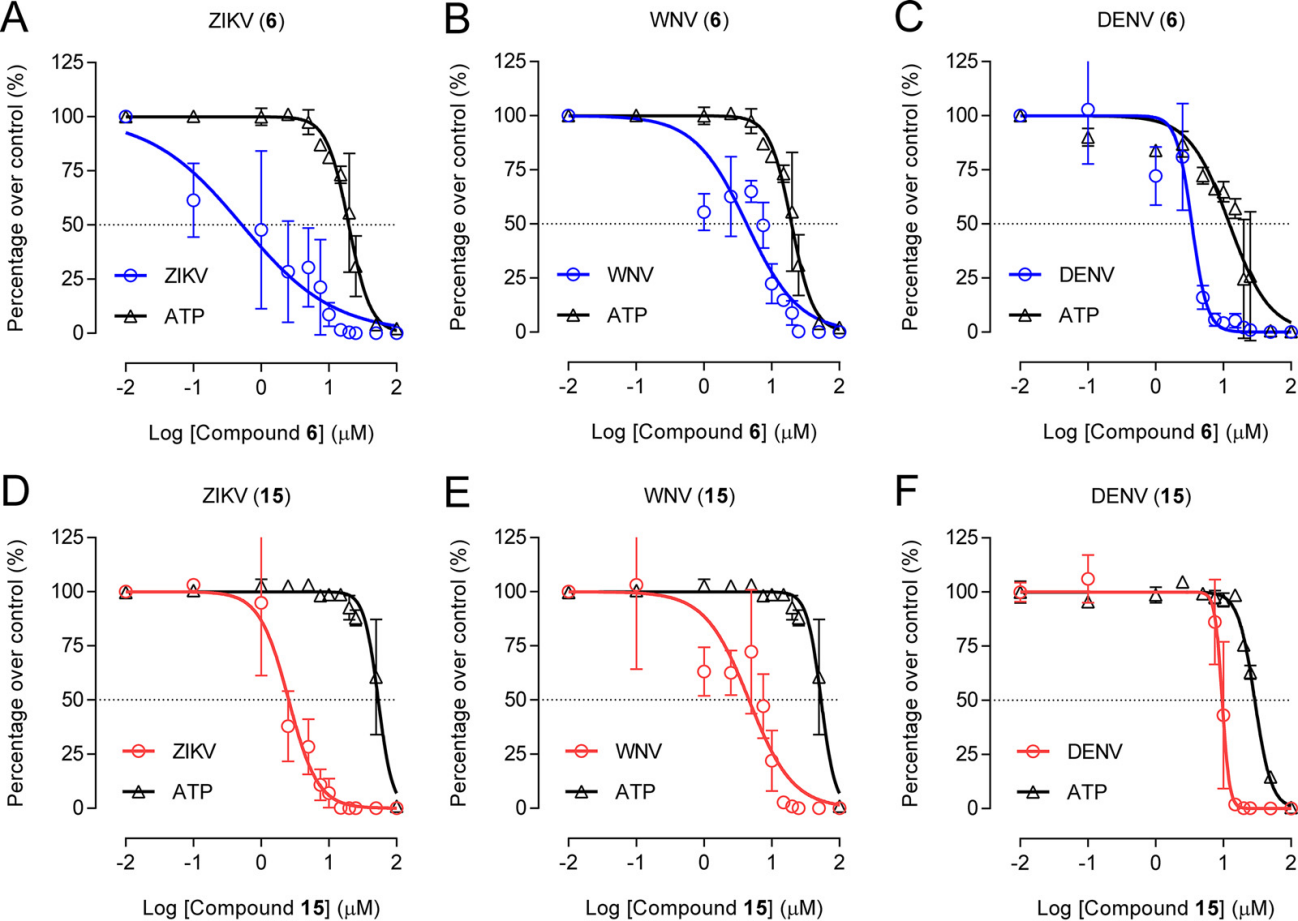
Antiviral activity of compounds 6 and 15 against medically relevant flaviviruses. (A to C) Dose-response curves of ZIKV (A), WNV (B), and DENV-2 (C) against compound 6. (D to F) Dose-response curves of ZIKV (D), WNV (E), and DENV-2 (F) against compound 15. Vero cells were infected (MOI of 1 PFU/cell) and treated with increasing amounts of the compounds, and virus yield in supernatant was determined at 24 hpi (ZIKV and WNV) or 48 hpi (DENV-2). The cytotoxicity of the compounds was estimated in parallel by quantification of cellular ATP in uninfected samples at 24 h (A, B, D, and E) or 48 h (C and F) (*n *=* 2 to 4*). Dashed lines denote a 50% reduction. Data are expressed as mean ± SD.

The antiviral efficacy of compounds 6 and 15 was analyzed in cultured cells. For this purpose, ZIKV and two medically relevant mosquito-borne flaviviruses, namely, West Nile virus (WNV) and dengue virus 2 (DENV-2), were selected (Fig. 3A to F). The two compounds exhibited good antiviral potencies with half-maximal effective concentrations (EC_50_s) within the low-micromolar range for all the flaviviruses assayed, these values being lower for ZIKV (Table 1). As an estimation of the safety of the compounds, the cytotoxicity was analyzed by calculating in parallel the half-maximal cytotoxic concentration (CC_50_) in each case (Fig. 3A to F and Table 1). These data were used to assess the relative effectiveness of the drug in inhibiting viral replication compared to inducing cytotoxicity by calculation of the selectivity indexes (SIs) for each compound and virus combination (Table 1). SIs ranged from 3.0 to 39.4, depending on each virus and compound, revealing that, although the relationship between efficacy and safety of the compounds was good for all the flavivirus tested, it was markedly higher for ZIKV (39.4 and 20.7 for compounds 6 and 15, respectively). Overall, these results support the antiviral activity of compounds 6 and 15 against ZIKV and other pathogenic flaviviruses.

**TABLE 1:** Selectivity indexes of compounds 6 and 15 for ZIKV, WNV, and DENV-2

Compound	CC_50_^*a*^ (μM) at:		EC_50_^*b*^ (μM) of:			SI^*c*^ (CC_50_/EC_50_) of:		
	24 h	48 h	ZIKV	WNV	DENV-2	ZIKV	WNV	DENV-2
TCMDC-143406 (compound 6)	19.7 ± 2.2	12.3 ± 2.1	0.5 ± 0.3	4.3 ± 0.9	3.4 ± 0.5	39.4	4.6	3.6
TCMDC-143215 (compound 15)	53.7 ± 4.2	29.2 ± 1.1	2.6 ± 0.5	4.6 ± 1.4	9.6 ± 0.9	20.7	7.1	3.0

a Half-maximal cytotoxic concentration (CC_50_) is the concentration that results in the reduction of 50% of the amount of cellular ATP after 24 or 48 h of treatment of uninfected cells. Data are expressed as mean ± SD (*n* = 2 to 4).

b Half-maximal effective concentration (EC_50_) is the concentration of drug at which virus yield (MOI of 1 PFU/cell) is inhibited by 50%. Virus yield was determined by plaque assay 24 hpi for WNV and ZIKV and 48 hpi for DENV-2. Data are expressed as mean ± SD (*n* = 2 to 4).

c Selectivity index (SI) is the ratio between CC_50_ and EC_50_. CC_50_ after 24 h of treatment was used to calculate SI for ZIKV and WNV. CC_50_ after 48 h of treatment was used to calculate SI for DENV-2.

### Mechanism of action.

**FIG 4:**
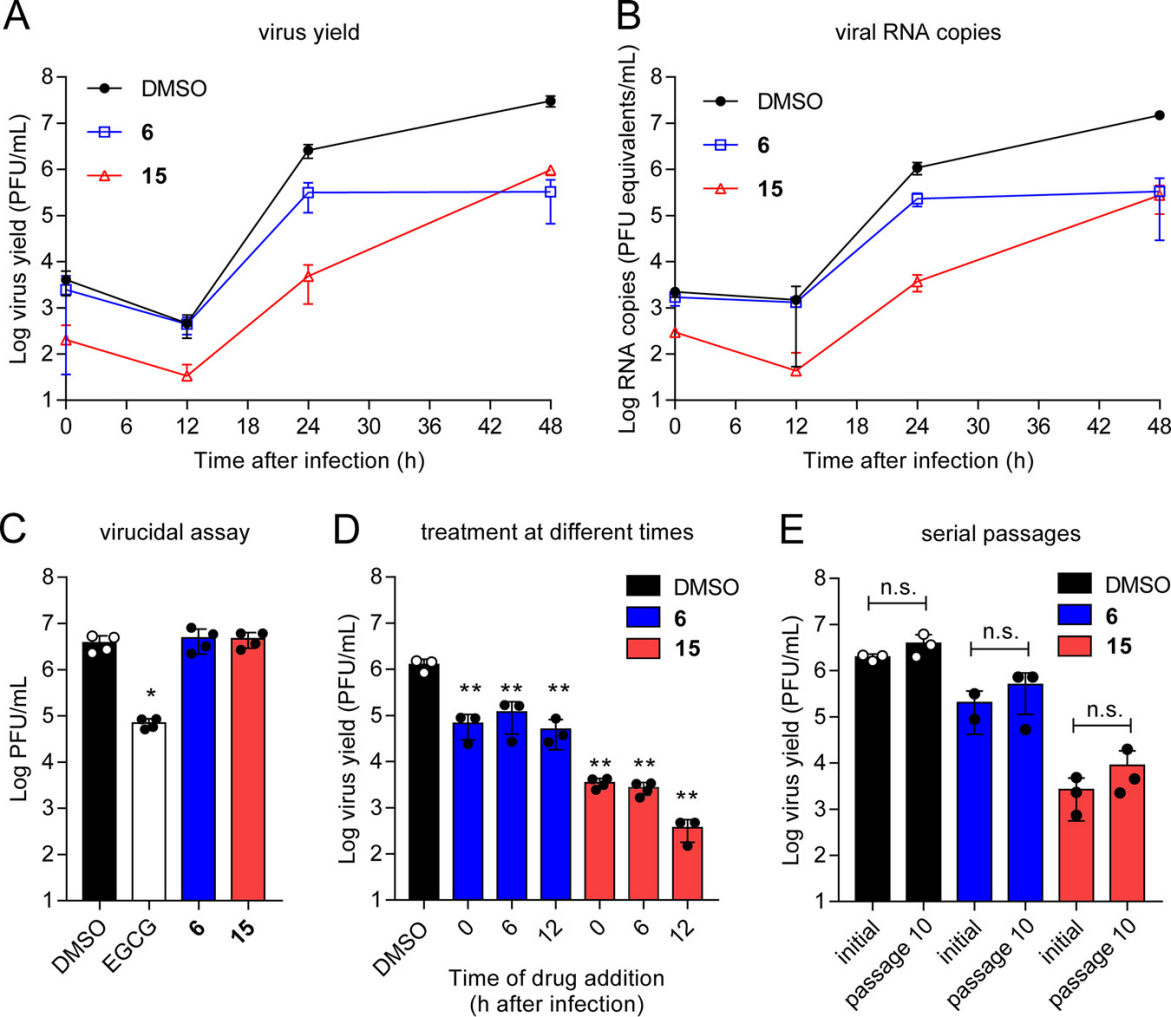
Effect of compounds 6 and 15 on ZIKV infection. (A) Inhibition of ZIKV production by compounds 6 and 15. Vero cells were infected with ZIKV (MOI of 1 PFU/cell) and treated with drug vehicle (DMSO), compound 6 (15 μM), or compound 15 (15 μM), and virus yield in supernatant was determined by plaque assay at the indicated times postinfection (*n* = 3). (B) Quantification of viral RNA in the supernatant of infected cultures in samples infected and treated as in panel A (*n* = 3). (C) Lack of virucidal effect of compounds 6 and 15. ZIKV (∼4 × 10^6^ PFU) was treated with drug vehicle (DMSO), EGCG (5 μg/ml), compound 6 (15 μM), or compound 15 (15 μM) for 1 h at 37°C in culture medium. Then, the infectivity in each sample was determined by plaque assay. EGCG was included in the experiments as a positive-control compound with virucidal activity (*n* = 4). (D) Time-of-addition experiments of compounds 6 and 15. Vero cells were infected (MOI of 1 PFU/cell) and treated with drug vehicle (DMSO), compound 6 (15 μM), or compound 15 (15 μM) from 0, 6, or 12 h after virus inoculation. Virus yield was determined by plaque titration at 24 hpi (*n* = 3 to 4). (E) Inhibition profiles of ZIKV after 10 serial passages in the presence of compounds 6 and 15. Vero cells were infected (MOI of 1 PFU/cell) with ZIKV stock (initial population) or the viral populations resultant from three-independent series of 10 passages (passage 10) in the presence of the compound 6 (15 μM) or compound 15 (15 μM). Virus yield was determined at 24 hpi by plaque assay (*n* = 2 to 3). *P* values were calculated using Dunnet’s test for pairwise comparisons of multiple treatment groups with a single control group in panels C and D or Tukey’s test for pairwise comparisons of the mean of each group with the mean of every other group in panel E. Asterisks denote statistically significant differences between control group (DMSO) and treatments. *, *P* < 0.05; **, *P* < 0.005; n.s., nonstatistically significant differences between groups. Data are expressed as mean ± SD. Points indicate independent biological replicates.

Given the good potency of compounds 6 and 15 against ZIKV, their antiviral activity was further characterized by different techniques. For each of the compounds, these experiments revealed a similar reduction in the production of the viral progeny either assessed by titration of infectious virus (Fig. 4A) or quantification of viral RNA (Fig. 4B). To exclude a direct effect of the compound on the infectivity of ZIKV, the potential virucidal effect of the compounds was evaluated (Fig. 4C). Incubation of the viral particles with compounds 6 or 15 did not reduce the infectivity, supporting that the inhibitory effect of the compounds was not related to a virucidal effect. On the contrary, (−)-epigallocatechin gallate (EGCG), included in the assay as a positive control due to its known virucidal effect against ZIKV (21, 22), significantly reduced the infectivity of ZIKV (Fig. 4C). To identify the viral step affected by the compounds, we performed a time-of-addition assay in which compound 6 or 15 was added at 0, 6, or 12 h relative to infection (Fig. 4D). In this assay, the potency of the inhibition of each compound was similar, even when added up to 12 h after infection. This suggests that the compounds acted during viral replication, which was compatible with the mechanism expected for RdRp inhibitors. Considering that the selection of resistant variants is a major determinant of the failure of direct-acting antivirals, ZIKV was subjected to 10 serial passages in the presence of each of the compounds. No significant increase in the resistance to the treatment was observed in viral populations propagated in the presence of compound 6 or 15 compared to viral populations passaged in the absence of the compounds (Fig. 4E). Nucleotide sequencing of the genomic region encoding full NS5 showed no nucleotide substitutions in those populations passaged in the presence of the compounds.

## DISCUSSION

The explosive emergence of ZIKV during the 2015 to 2016 epidemic resulted in a huge effort of the scientific community in the search for strategies to fight this long-known but, until recently, neglected pathogen. However, there is still no vaccine or antiviral therapy licensed (23). Due to the fundamental role of NS5 in the replicative cycle of ZIKV and its unique enzymatic activities, this protein is a main target for the development of specific antiviral treatments with direct action and low toxicity (24). Several studies have been reported on the antiviral effect of nucleoside analogues inhibitors (NIs) against the RdRp activity of NS5 (25–28). However, NI-based therapies still present challenges. One of them is that they must be administered in prodrug forms to be converted into the active-form triphosphate inside the cells, thus complicating their mode of action. Alternatively, nonnucleoside analogue inhibitors (NNIs) that act on allosteric regulation sites constitute an interesting therapeutic option, either alone or searching for combined therapies with NIs (29–31). Here, we have employed our recently developed high-throughput screening platform to evaluate the potential inhibitory effect of different compounds against the ZIKV NS5 RdRp domain. After screening an open-source library of compounds envisioned as antikinetoplastid parasites, two hits (compounds 6 and 15) that totally abrogated fluorescence-associated activity of ZIKV NS5 were identified. It could be unusual to find that antikinetoplastid candidates constitute RdRp inhibitors, but there are examples of other antiparasitic drugs that also exert antiviral activities. For instance, ivermectin, a broadly used antihelminthic drug, is also an inhibitor of flavivirus NS3 ATPase activity (32). Moreover, drug-repurposing studies have pointed to the anti-ZIKV properties of other antiparasitics, antibiotics, antiprotozoals, or antifungals (29, 31, 33, 34). When tested against other RdRps from other viral genera, it was observed that compound 6 fully inhibited the activity of the RdRps from HCV and FMDV, claiming the broad-spectrum activity of this compound. On the contrary, the inhibitory activity of compound 15 against HCV and FMDV RdRps was reduced compared to ZIKV RdRp. The kinetic data obtained in the fluorescence assays indicated that compounds 6 and 15 exerted noncompetitive inhibition and antagonistic activity in compound combination assays. This behavior is compatible with both inhibitors binding to the same allosteric regulatory site. In fact, previous studies have described other ZIKV allosteric inhibitors that bind near the active site (16) and identified at least an allosteric pocket (N-pocket) in the NS5 RdRp domain of DENV (35) that is also conserved in ZIKV (30), with which it shares 68.1% of identity. However, we have no experimental data to support whether that this allosteric pocket constitutes the target of these compounds, and further work should be performed to clearly identify the binding site of these inhibitors.

Compounds 6 and 15 inhibited ZIKV multiplication in cultured cells showing good potencies and high selectivity. Therefore, their antiviral efficacy was also extended to other pathogenic flaviviruses (WNV and DENV). Although the potency observed was lower than for ZIKV, the conservation of the antiviral activity against these three flaviviruses tested suggests the potential of these compounds for the future development of pan-flavivirus inhibitors. Compared to the polymerization assays, the EC_50_ values obtained in cell culture experiments were about 10-fold lower for each compound. This could be explained by the differences between *in vitro* polymerization assays and cell culture infection assays because the fluorescence-based method only includes the RdRp domain and was mainly designed to exploit the ability of ZIKV polymerase to initiate RNA synthesis *de novo*. Notably, the differences in the potency between compounds 6 and 15 were about 5-fold in both biochemical (IC_50_) and infectious assays (EC_50_), thus reinforcing the validity of this simple approach to identify and characterize antiviral candidates. Quantification of viral RNA and time-of-addition experiments showed that the mechanism of inhibition of ZIKV infection was compatible with that expected for RdRp inhibitors. In addition, no resistant mutants were found after 10 serial passages in the presence of the compounds, which could be indicative of a high fitness cost of resistance against these inhibitors. Considering that resistance variants constitute one of the major causes of failure of antiviral therapies (36, 37), the lack of resistance would be beneficial for potential therapeutic approaches. In this regard, the experiments here reported were mainly aimed to evaluate whether these compounds were highly prone to resistant variant selection, and considering our results, this may not be the case. Complementary approaches using more stringent conditions of the inhibitors throughout the successive viral passages might lead to the emergence of a resistant mutant (38), although, in some cases, the isolation of mutants is not an easy task, and the resistance to inhibitors targeting the viral RdRp could even emerge by mutations in other viral proteins (39). Whereas the results obtained with cellular and biochemical assays were compatible with inhibitors targeting viral replication such as RdRp inhibitors, due to the lack of resistant mutant selection to identify the specific target, we cannot exclude that other mechanisms could also contribute to the cellular antiviral activity of the compounds in cell cultures. Thus, further experiments should be performed to better study the ability of these inhibitors to produce resistant variants.

In conclusion, we have assessed the usefulness of our previously designed high-throughput screening platform of anti-ZIKV NS5 by challenging it against a library of compounds without predicted antiviral activity. Two compounds (6 and 15) that completely abrogated the polymerase activity of ZIKV RdRp by a noncompetitive inhibition mechanism were identified. These compounds exhibited good antiviral potencies against ZIKV and other related flaviviruses, becoming potential leads for further research into broad-spectrum antiflavivirus drugs.

## MATERIALS AND METHODS

### Reagents.

From Applichem, the following reagents were obtained: MnCl_2_, 1,4-dithiothreitol (DTT), bovine serum albumin (BSA), and Tris base. Stock solutions of ATP and SYTO-9 were purchased from Thermo Fisher. poly(A) was obtained from Amersham Biosciences. (−)-Epigallocatechin gallate (EGCG) and dimethyl sulfoxide (DMSO) ≥99.9% were obtained from Sigma. Antikinetoplastid chemical boxes, used during the initial screening, were provided by GlaxoSmithKline (GSK) under material transfer agreement no. MTARG566. TCMDC-143406 (compound 6) [4-(5-amino-4-cyclohexyl-3-isopropyl-1H-pyrazol-1-yl)-6-(pyridin-2-yl)-1,3,5-triazin-2-amine; PubChem compound identifier (CID) 91800701] and TCMDC-143215 (compound 15) {1-[6-(pyridin-2-yl)-2-(*p*-tolylamino)pyrimidin-4-yl]piperidin-3-ol; PubChem CID 91800580}, the latter as an equimolar mixture with trifluoroacetic acid, were chemically synthesized in dry powder by Enamine. Ltd. They were dissolved in DMSO, aliquoted, and frozen at −80°C for further use.

### Library screening and polymerase activity assays.

Expression and purification of recombinants FMDV 3D, HCV NS5b, and ZIKV NS5 RdRps were carried out as previously described (18, 40). Real-time fluorescence-based polymerization assays using a 96-well format were carried out according to the protocol previously developed (18) with the following variations. Compound library screening of the three open-access antikinetoplastid chemical boxes (in total, 592 compounds [19]) were carried out using ready-to-use 96-well flat-bottom plates (Corning) provided by GSK in triplicate. Every individual well of plates contained 0.5 μl of each compound (10 mM in DMSO). The assay was initiated by the addition of 49.5 μl of standard reaction solution [50 mM Tris-HCl, pH 7.5, 2.5 mM MnCl_2_, 500 μM ATP, 20 μg/ml poly(U), 0.1 mg/ml BSA, 0.25 μM SYTO-9, and 250 nM recombinant ZIKV RdRp motive] using an automatic 8-multichannel pipette, and plates were subjected to analysis in less than 1 min. The delay between initial and final well was negligible considering that the increase in fluorescence takes about 5 min to be detectable (Fig. 1B). Thus, the final concentration of each compound in the reaction was 100 μM. At least four reactions in the presence of 1% DMSO were carried out in each plate used as positive control. Fluorescence kinetics were recorded over 20 min at 30°C using a FLUOstar Optima fluorimeter (BMG Labtech). Only compounds that exhibited total inhibition of the fluorescence-associated activity compared to a DMSO control during the whole course of the reaction were considered hits. In the rest of the biochemical assays described below (IC_50_ determination, characterization of the inhibition kinetics, and compound combination assays), the reaction mix (except polymerase) was first added to the plates, then compounds were added to each well, and the polymerization reaction was initiated by addition of the polymerase.

For IC_50_ determination of both compounds 6 and 15, fluorescence-based polymerization assays were performed with increasing concentrations of each inhibitor using 3 μg/ml poly(U) and 1,500 μM ATP as the substrates, keeping the rest of the components as the aforementioned standard reaction solution. Relative activity values were determined as the velocity of polymerization recorded from minute 10 to minute 20 of the reaction.

To determine the inhibition kinetics of compounds 6 and 15, real-time fluorescence-based polymerization assays using standard solution were performed in the presence of variable ATP (200 to 2,250 μM) as the substrate and either compound 6 (10 and 20 μM) or compound 15 (40 and 70 μM) as inhibitor. To determine the mechanism of inhibition, Lineweaver-Burk plots were constructed from polymerization velocity data calculated from minute 10 to minute 20 of the reaction.

Evaluation of simultaneous administration effect of compounds 6 and 15 was achieved by performing fluorescence-based polymerization assays in the presence of different mixtures of compounds 6 and 15 doses (1.5 to 6 μM and 7.5 to 30 μM, respectively). These experiments were performed by adding 0.5 μl of the corresponding mixture diluted in DMSO to 49.5 μl of standard reaction solution. The polymerization velocity values recorded during the second 10 min of the reaction were used to calculate the combination index plot (CI) (41) utilizing the CompuSyn software (http://www.combosyn.com/feature.html).

Fluorescence-based polymerization assays with recombinant HCV NS5b and ZIKV NS5 RdRps in the presence of compounds 6 and 15 were conducted as described above. The standard reaction solution was used in the presence of 400 nM of the specific recombinant RdRp and 100 μM of each inhibitory compound. The presence of either 1% DMSO or 100 μM trifluoroacetic acid in real-time polymerization assays did not show any inhibitory effect as judged by control experiments (data not shown). FMDV three-dimensional RdRp polymerization assays were carried out with 400 nM recombinant polymerase and 100 μM each inhibitory compound using the reaction conditions described previously (18).

### Cells, viruses, infections, and virus titrations.

Infectious virus manipulations were conducted in biosafety level 3 facilities. The origin of ZIKV PA259459, WNV NY99, and DENV-2 isolates has been previously described (42). All infections were performed on Vero CCL-81 cells (ATCC). Procedures for infections and virus titrations in semisolid agar medium have been previously described (42, 43). ZIKV and WNV titers were determined 24 h postinfection (hpi). DENV-2 titers were determined 48 hpi. The multiplicity of infection (MOI) was expressed as PFU/cell and is indicated in the figure legends.

### Drug treatments.

Unless otherwise specified, compounds were added after 1 h of incubation of the viral inoculum with the cell monolayers to allow virus adsorption and entry and were maintained throughout the rest of the assay. Control cells were treated in parallel with the same amount of drug vehicle (DMSO). Cell viability was estimated in uninfected cells by ATP measurement using the CellTiter-Glo luminescent cell viability assay from Promega.

### Quantitative PCR and nucleotide sequencing.

ZIKV RNA was extracted from culture supernatants using QIAmp viral RNA minikit (Qiagen) and a QIAcube apparatus (Qiagen). Viral RNA was quantified by real-time fluorogenic reverse transcriptase PCR (RT-PCR) (44). Data are expressed as PFU equivalents/ml by comparison with previously titrated samples. The cDNA encoding the ZIKV NS5 region was amplified by RT-PCR, and the nucleotide sequence was determined by automated nucleotide sequencing (Macrogen, Madrid, Spain).

### Data analysis.

Data are presented as mean ± standard deviation (SD). The number of independent biological replicates (*n*) analyzed is indicated in the figure legends. All analyses were performed using Prism 7 for Windows (GraphPad Software, Inc.). Dose-response curves were calculated by adjusting the sigmoidal log(inhibitor) versus normalized response (variable-slope) equation. Means were compared using one-way analysis of variance (ANOVA) corrected for multiple comparisons with Dunnet’s (pairwise comparisons of multiple treatment groups with a single control group) or Tukey’s correction (pairwise comparisons of the mean of each group with the mean of every other group). Statistically significant differences are denoted by asterisks. *, *P < *0.05; **, *P < *0.005; n.s., nonstatistically significant differences between groups.
